# Recommendations for Uniform Variant Calling of SARS-CoV-2 Genome Sequence across Bioinformatic Workflows

**DOI:** 10.3390/v16030430

**Published:** 2024-03-11

**Authors:** Ryan Connor, Migun Shakya, David A. Yarmosh, Wolfgang Maier, Ross Martin, Rebecca Bradford, J. Rodney Brister, Patrick S. G. Chain, Courtney A. Copeland, Julia di Iulio, Bin Hu, Philip Ebert, Jonathan Gunti, Yumi Jin, Kenneth S. Katz, Andrey Kochergin, Tré LaRosa, Jiani Li, Po-E Li, Chien-Chi Lo, Sujatha Rashid, Evguenia S. Maiorova, Chunlin Xiao, Vadim Zalunin, Lisa Purcell, Kim D. Pruitt

**Affiliations:** 1 National Center for Biotechnology Information, National Library of Medicine, National Institutes of Health, Bethesda, MD 20894, USA; connorrp@ncbi.nlm.nih.gov (R.C.); jamesbr@ncbi.nlm.nih.gov (J.R.B.); jonathan.gunti@nih.gov (J.G.); jinyu@ncbi.nlm.nih.gov (Y.J.); kskatz@ncbi.nlm.nih.gov (K.S.K.); andrey.kochergin@nih.gov (A.K.); xiao2@ncbi.nlm.nih.gov (C.X.); vadim.zalunin@nih.gov (V.Z.); 2 Bioscience Division, Los Alamos National Laboratory, Los Alamos, NM 87545, USA; migun@lanl.gov (M.S.); pchain@lanl.gov (P.S.G.C.); bhu@lanl.gov (B.H.); po-e@lanl.gov (P.-E.L.); chienchi@lanl.gov (C.-C.L.); 3 American Type Culture Collection, Manassas, VA 20110, USA; dyarmosh@atcc.org (D.A.Y.); rbradford@atcc.org (R.B.); srashid@atcc.org (S.R.); 4 BEI Resources, Manassas, VA 20110, USA; 5 Galaxy Europe Team, University of Freiburg, 79085 Freiburg, Germany; maierw@informatik.uni-freiburg.de; 6 Clinical Virology Department, Gilead Sciences, Foster City, CA 94404, USA; ross.martin@gilead.com (R.M.); jiani.li23@gilead.com (J.L.); evguenia.svarovskaia@gilead.com (E.S.M.); 7 Deloitte Consulting LLP, Rosslyn, VA 22209, USA; cocopeland@deloitte.com (C.A.C.); flarosa@deloitte.com (T.L.); 8 Vir Biotechnology Inc., San Francisco, CA 94158, USA; jdiiulio@vir.bio (J.d.I.); lpurcell@vir.bio (L.P.); 9 Eli Lilly and Company, Indianapolis, IN 46225, USA; ebertpj@lilly.com

**Keywords:** SARS-CoV-2, variant calling

## Abstract

Genomic sequencing of clinical samples to identify emerging variants of SARS-CoV-2 has been a key public health tool for curbing the spread of the virus. As a result, an unprecedented number of SARS-CoV-2 genomes were sequenced during the COVID-19 pandemic, which allowed for rapid identification of genetic variants, enabling the timely design and testing of therapies and deployment of new vaccine formulations to combat the new variants. However, despite the technological advances of deep sequencing, the analysis of the raw sequence data generated globally is neither standardized nor consistent, leading to vastly disparate sequences that may impact identification of variants. Here, we show that for both Illumina and Oxford Nanopore sequencing platforms, downstream bioinformatic protocols used by industry, government, and academic groups resulted in different virus sequences from same sample. These bioinformatic workflows produced consensus genomes with differences in single nucleotide polymorphisms, inclusion and exclusion of insertions, and/or deletions, despite using the same raw sequence as input datasets. Here, we compared and characterized such discrepancies and propose a specific suite of parameters and protocols that should be adopted across the field. Consistent results from bioinformatic workflows are fundamental to SARS-CoV-2 and future pathogen surveillance efforts, including pandemic preparation, to allow for a data-driven and timely public health response.

## 1. Introduction

Genomic sequencing has been used to address public health crises for more than a decade [1]. The Center for Disease Control noted its utility in screening for heritable and infectious disease as early as 2014, and now it is applicable to public health applications ranging from tracking a specific pathogen to developing vaccines against them [2]. Likewise, during the COVID-19 pandemic, there was an unprecedented level of sequencing of Severe Acute Respiratory Syndrome Coronavirus 2 (SARS-CoV-2) genomes. There have been over 15.1 million SARS-CoV-2 sequence submissions from over 10.2 million samples in the National Institute of Health (NIH), National Library of Medicine (NLM), National Center for Biotechnology Information (NCBI) open access repositories GenBank [3], and the Sequence Read Archive (SRA) [4] as of 1 December 2023. While many public health agencies and their partners around the world have been sequencing the virus at unprecedented levels and making the data available via public sequence repositories like GISAID and GenBank [5], the useful integration of this data from diverse data generators critically depends on consistent analytical methods and transparency in how the data was processed prior to public release [6,7]. For example, in mid-2020, single mutations in the spike protein distinguished different clades of the virus [8]. Any artifactual differences due to upstream processing could have significant impacts on epidemiologic interpretation, the public health response, or simply add to public confusion around the pandemic. Finally, regulatory agencies including the Food and Drug Administration (FDA) required submission of Next Generation Sequencing (NGS) data and the interpretation by pharmaceutical companies for new drug applications and monitoring of the virus throughout the therapeutic lifecycle. Therefore, accurate and reproducible detection of mutations in SARS-CoV-2 sequencing data is critical for effective management of the SARS-CoV-2 pandemic, and the development of vaccines and antiviral drugs.

A typical bioinformatic workflow for generating SARS-CoV-2 genome starts with identifying mutations, which can be either Single Nucleotide Polymorphisms (SNPs) or Insertions/Deletions (Indels), which are inferred based on raw NGS data mapping to a reference sequence [9]. One of the resulting products of this comparison is a consensus genome, which is then used for many downstream applications, including phylogenomic studies and tracking genomic variants, all of which are critical for public health decision-making. Thus, understanding the differences between different bioinformatic workflows is critical, as downstream analytics, like identifying SARS-CoV-2 variants, typically assume consistency and error-free genomes, which is usually not true for sequences across the full public dataset. Notably, the assumptions and constraints relevant to workflows that call these mutations and consensus genomes differ from one tool to another. Despite millions of genomes having been deposited in public databases by thousands of institutions using multiple different tools, there has not yet been a comprehensive analysis of bioinformatic workflows that call mutations and generate consensus genomes from raw reads. Such comparison of processing workflows, which include read aligners, variant callers, and sequencing instruments, has been assessed by various studies for human genome data [10,11,12,13,14,15,16], resulting in best practices for germline [17] and somatic variant detections [18]. While there have been several efforts to optimize workflows specifically for SARS-CoV-2 thus far [19], including those for which representatives participated in the current study, general recommendations or best practices remain absent during the last pandemic, and are still needed for any future ones.

To address this issue, as part of the National Institutes of Health’s (NIH) Accelerating COVID-19 Therapeutic Interventions and Vaccines Tracking Resistance and Coronavirus Evolution (ACTIV TRACE) initiative [20], the Foundation for NIH (FNIH) convened seven groups that have developed their own bioinformatic workflows across government, industry, and academia to compare and contrast several different approaches to SARS-CoV-2 NGS data analysis, thus capturing a variety of use cases. The goal of the collaboration was to identify conditions under which variant call results were likely to be agreed upon across workflows and sequencing platforms, using real-world data. Accordingly, the group first identified a shared test dataset against which the results of their respective workflows were compared. Then, all the differences between workflows were used to identify specifications under which consistent results across workflows could be assured. Importantly, the focus of the effort was to identify cross industry, academic, and government guidelines for SARS-CoV-2 deep sequencing analysis.

## 2. Results

### 2.1. Datasets

In order to focus this study on workflows, two datasets were provided to each of seven participating teams, including BEI Resources, Galaxy, Gilead Sciences, Los Alamos National Laboratory (LANL), Eli Lilly and Company, National Center for Biotechnology Information (NCBI, NIH), and Vir Biotechnology. The sequence records were retrieved from the NCBI SRA, which contains raw sequencing data, and afforded these teams the ability to run independent workflows on the same raw data. The two datasets were provided to the teams, and were selected based on the availability of Illumina and Oxford Nanopore (ONT) data sequenced from same sample. Dataset 1 consisted of 413 sequence records, representing 155 samples, and was used to conduct the initial analysis comparing each workflow’s protocol. These samples were from February 2020 to May 2021, and generally represented the Alpha lineage. A subsequent test dataset, Dataset 2, consisted of 419 sequence records, representing 210 samples, and was used to compare concordance across workflows. Dataset 2 sequences were deposited at the start of the Omicron wave, representing samples obtained from April 2021 to April 2022. There are six BioSamples and twelve accessions (two different sequencing platforms per platform) that are included in both datasets. Datasets are available from https://github.com/ncbi/ACTIVTRACEvariants/ (accessed on 4 December 2023).

### 2.2. SARS-CoV-2 Bioinformatic Workflow Overview

**Figure 1:**
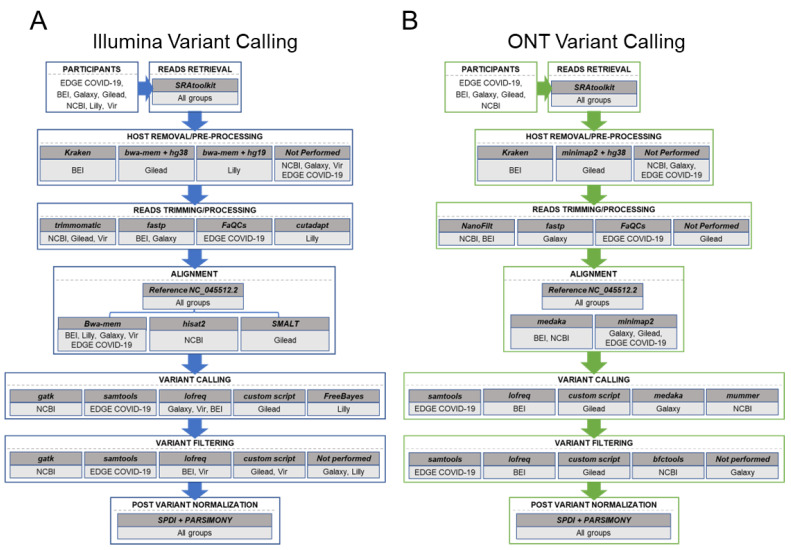
Flow chart of the platforms from each participating organization’s workflows at the time of analysis. Shown are the schematics for (**A**) Illumina platform variant calling and (**B**) Oxford Nanopore Technologies (ONT) variant calling. For each sequencing platform, the main steps of variant calling are captured in each box, including: read retrieval, host removal, read trimming, alignment, variant calling, variant filtering, and variant normalization. For each step, the software used by each workflow is noted.

The test dataset consists of genomes generated with Illumina and ONT. Illumina sequencing is primarily short-read, sequencing-by-synthesis platform [21], while ONT is a long-read technology with an electric current used to read the bases through a protein nanopore [22]. Each of these platforms varies in terms of accuracy and ability to identify rare base/insertion/deletions, which needs to be considered during the development of workflows and complicates the generation of comparable results across platforms. Variant calling workflows from academic, industry, and government groups follow a similar generic bioinformatics workflow for processing SARS-CoV-2 genomes (Figure 1). These steps include data retrieval from SRA, pre-processing (with NCBI’s workflow not doing primer-trimming, and NCBI, Galaxy, Vir, and LANL’s EDGE-COVID19 workflows not performing host-removal as part of the analyses at the time of analysis), QC of raw reads (with trimmomatic particularly common among Illumina workflows), alignment/reads mapping to a reference genome (with minimap2 being especially common among ONT workflows), variant calling, and variant filtering (post-processing). A more detailed comparison of each workflow’s software and parameters is summarized in Supplementary Table S1. Of note, in agreement with the variety of software available for each, we found a much greater diversity in software used for Illumina platform data (Figure 1A) than ONT (Figure 1B), further supporting the generalizability of our results.

### 2.3. Data Pre-Processing Impacts on Variant Calling

*Many workflows did not call same SNPs.* Initially, when all the reported SNPs and indels were considered, the workflows differed significantly, so a minimum Allele Frequency (AF: frequency of a specific SNP) of at least 15% was considered moving forward. Still, despite this threshold, the initial results showed less than 27% agreement for SNPs in Dataset1. Surprisingly, no indels were called by all seven of the workflows. 

**Figure 2:**
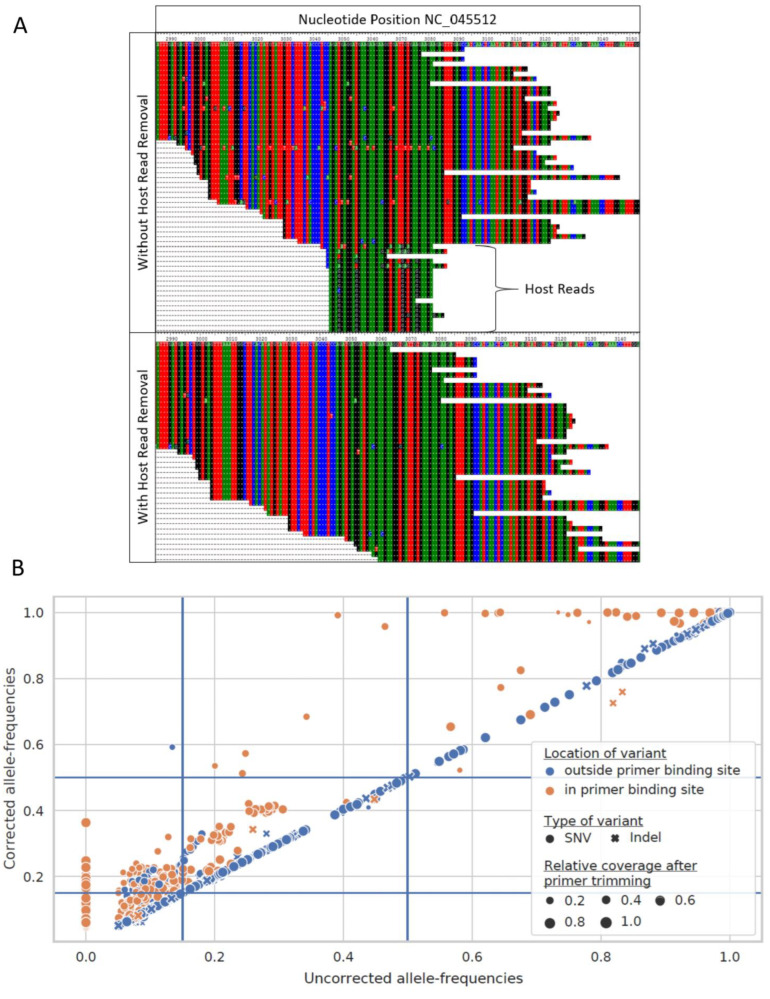
The impact of host contamination removal and primer trimming. (**A**) The removal of host reads from RNAseq SARS-CoV-2 sequencing result, SRA run SRR12245095, reduced the potential for false-positive variant calls. In the top panel, additional mutations were present in aligned reads between positions 3049–3076 of NC_045512 when host reads were not removed. After excluding host reads (bottom panel), reads containing the mutations were no longer observed. (**B**) Allele frequencies of variants called after trimming primer sequences from aligned reads (corrected allele frequencies) are plotted against allele frequencies of the same variants called without primer trimming (uncorrected allele frequencies). Primer trimming increases the allele frequencies of most within-primer binding sites variants. Blue lines represent the allele-frequency thresholds used in this study to filter variant calls (allele frequency; AF ≥ 0.15) and to call consensus variants (AF ≥ 0.5).

*Host RNAs when not removed can result in wrong mutation*. Although most depositors likely take steps to remove the host contaminate sequences from sequencing data, traces of human sequence data may still be present, and contribute to differences in variant calling results. We conducted an analysis across workflows, and determined that not all workflows were excluding possible human host-reads at the time of analysis. Detection of host read data was done via the SRA Taxonomy Analysis Tool (STAT) [23] and alignment to human reference. Figure 2A shows a number of spurious calls when host-read data is not removed; note the region between 3050 and 3090 in particular, with host-derived reads at the bottom, including some spurious variant calls. This results in an artificial reduction in the observed frequency of true calls, as the host reads identified in Figure 2A artificially inflate the frequency of the reference allele, as indicated by the matching color at each position. 

*Primer sequences, when not removed, can decrease the frequency of true variant calls*. All of the data that were analyzed as part of this paper, and most of the available SARS-CoV-2 genomes, were sequenced by amplifying overlapping regions across the genome. Thus, many of the downstream workflows include a step where the primers are trimmed from the reads before calling mutations, as untrimmed sequences could artificially influence the variant calling results, and thus agreement across our pipelines here. However, workflows differed significantly on how the primers were trimmed from the sequenced reads. As seen in Figure 2B, failure to trim primer sequence from reads results in a reduction in the reported frequency of many variants in primer binding sites. This can reduce variant frequency below the threshold for consideration, which in our case was 15%, or below the threshold for incorporation into a consensus sequence, which is typically 50%. Primer trimming increases the AFs of 61 within-primer binding site variants above the threshold for retaining them, and enables the calling of six additional consensus variants. Unfortunately, as was the case for the data retrieved for the dataset here, the information on the primer sets used for sequencing was not readily available, which complicated the analysis. While in some environments, the availability of high quality samples does not require de-hosting, as, in general, both host contaminant sequence removal and primer trimming are necessary to ensure accuracy and consistency of deep sequencing results.

### 2.4. A Parsimony Normalization Method to Standardize Variant Reporting across All Workflows Allowed Vis-a-Vis Comparison of Variants

The comparison of results across all seven workflows was also complicated by differences in how SNPs and indels are reported by each workflow, which was most pronounced in the case of indels. To address this, two steps were taken. First, the SPDI algorithm [24], used as part of the dbSNP (https://www.ncbi.nlm.nih.gov/snp/, accessed on 4 December 2023) and ClinVar (https://www.ncbi.nlm.nih.gov/clinvar/, accessed on 4 December 2023) databases at NCBI (as of the time of publishing), for human genetic variants, was adapted for use with SARS-CoV-2 deep sequencing. While the implementation of the SPDI algorithm greatly reduced the number of observed discrepancies due to formatting, especially for deletions (e.g., ATCG1CG vs. TCG2T vs. CG3-, all normalized to TCG2T), insertion differences remained (e.g., ATC2ATCTAG vs. C2CTAG vs. -3TAG). Second, to address the consistency in calling indels across workflows, a Parsimony algorithm was developed (see Section 4) to ensure the number of preceding and subsequent bases around an event were consistent, and that the events were left-aligned to a common starting position. Additionally, sequence records, for which less than 50% of the reference genome was covered or for which the average depth of coverage was less than 100X, were excluded, along with the analysis of single-end read data, since not all workflows supported this analysis. Finally, as many of the indel discrepancies that could not be resolved with the combination of SPDI and Parsimony approaches were found to be in homopolymer regions (of length at least 4 bp), the homopolymer regions were also excluded (Supplemental Figure S1). Together, these approaches help minimize the possibility that the remaining indel discrepancies were artifactual, and increased the likelihood that they represented genuine differences between workflows. This also highlights that simply cleaning data prior to processing is not sufficient to ensure broad workflow agreement in variant calling results.

### 2.5. Within-Sample Allele Frequency and Per-Position Depth of Coverage Determine the Consistency of Variant Calls across Workflows

**Figure 3:**
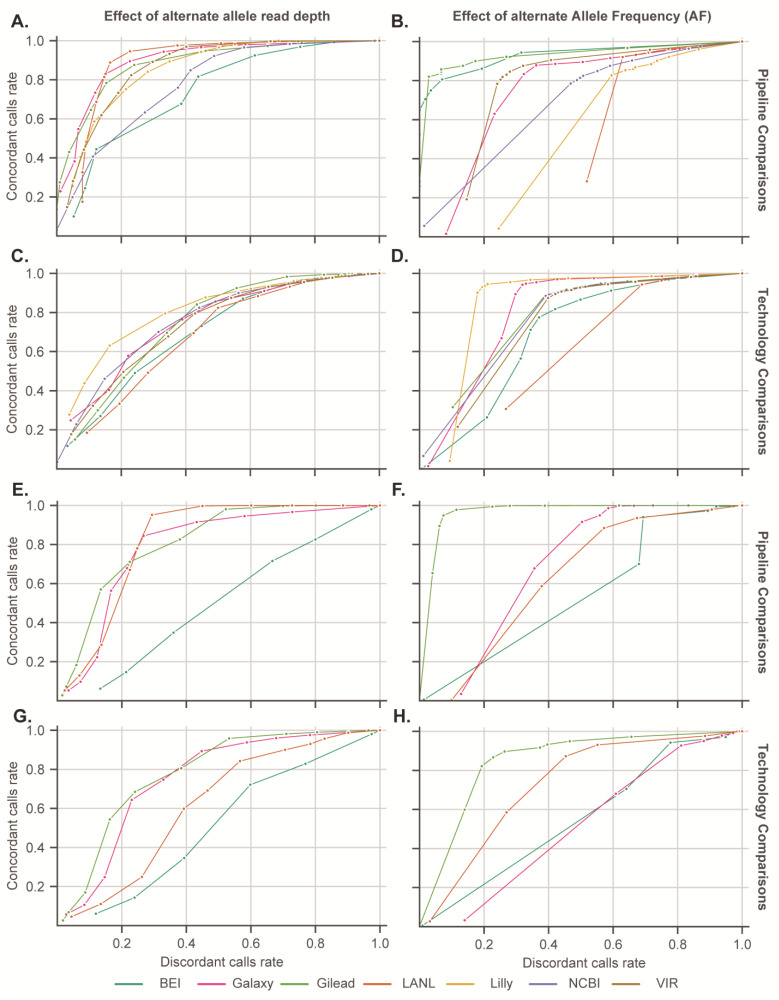
The effect of Alternate Allele Depth and Alternate Allele Frequency on variant calling agreement across workflows and platforms. For each panel, calls made by all but one workflow (**A**–**D**) or both platforms (**E**–**H**) were considered true-positives, while calls made by only a single workflow (or technology) were considered false-positives, thus the ROC AUCs cannot be directly compared between groups. For the right panels, points represent an Allele Frequency (AF) cut-off of 1 at the lower-leftmost point, and the cut-off decreases by 0.1 along the length of the line. For the left panels, the points represent a minimum Alternate Allele Depth (AltDP) going from 4,000 at the lower-left most point to 10 along each line. (**A**,**B**) Impact of AltDP and AF, respectively, on Illumina workflow accuracy and specificity across workflows. (**C**,**D**) Impact of AltDP and AF, respectively, on Illumina workflow accuracy and specificity across platforms. (**E**,**F**) Impact of AltDP and AF, respectively, on ONT workflow accuracy and specificity across workflows. (**G**,**H**) Impact of AltDP and AF, respectively, on ONT workflow accuracy and specificity across platforms.

In order to remain agnostic regarding the software used in each workflow, and with the aim of optimizing agreement between workflow results, as there are no ground-truth variant calling results for our dataset, the agreement between workflows and across platforms was assessed via Receiver-Operating Characteristic (ROC) analysis. For this purpose, variants identified by five or more workflows were considered true-positives, while calls made by fewer than five workflows were considered a false-positive. For this reason, the area under the ROC curves (ROC AUC) cannot be directly compared between groups. The rationale is that if a variant call is accurate, it should be found by any workflow (or platform), regardless of the implementation specifics. However, the ROC curves can identify the inflection point across parameter settings and suggest settings that maximize the true-positive results (as defined above) within each workflow or platforms, while minimizing the false-positive variant calls. For the assessment of agreement between sequencing platforms, variants found on both platforms were considered a true positive, while variants found by a single platform were considered a false-positive. Thus, false-positives here represent calls that were not common to workflows or sequencing platforms, but that does not necessarily mean they are incorrect, only that very few of the workflows considered here found them. Figure 3 illustrates ROC analyses of the effects of minimum Alternate Allele Depth of Coverage (ALTDP) and minimum AF on variant calling agreement. (Figure 3A–D) shows agreement across workflows, while Figure 3E–H considers agreement across sequencing platforms. The most sensitive discriminator, the parameter for which a sharp inflection point was identified for most workflows, was found to be ALTDP (Figure 3A,C), while the best discriminator for results between platforms was found to be AF, as depicted in (Figure 3F,H). Accordingly, a minimum ALTDP of 50 and a minimum AF of 50% was used for subsequent analyses, as these parameters minimize disagreement across workflows and sequencing platforms while retaining the maximum number of shared variant calls. Additionally, as overall read depth (DP) was also found to perform well for comparison of results across workflows, a minimum DP of 100 was included, which is further supported by prior work on synthetic data identifying minimum depth of coverage for calling variants at low-frequencies [25].

### 2.6. Filtering for Highly Supported Variant Calls Supports Cross-Workflow and Cross-Platform Agreement

**Figure 4:**
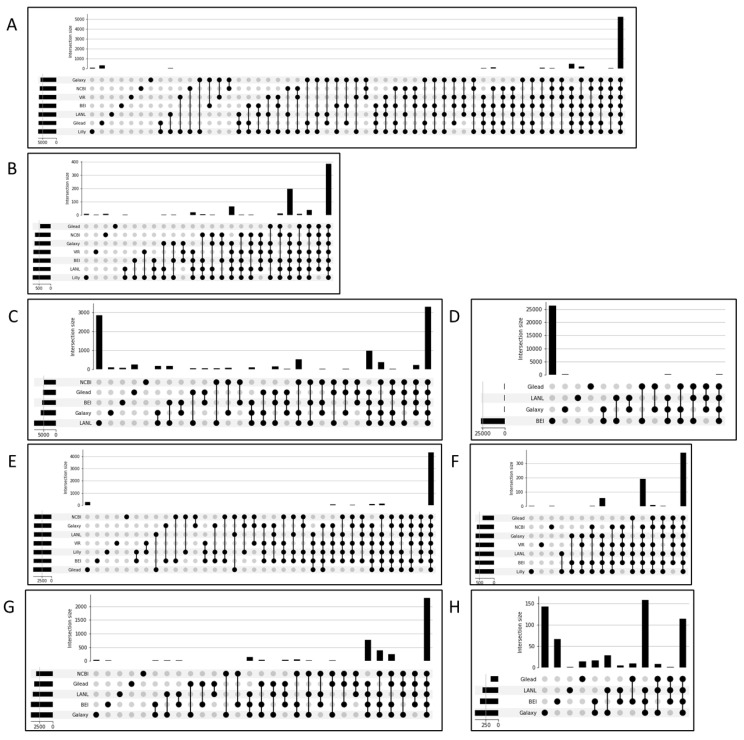
Agreement across workflows with and without recommended parameters. (**A**–**D**) Agreement across workflows, without recommended parameters. (**E**–**H**) Agreement across workflows, with recommended parameters. (**A**,**E**) Agreement on Illumina SNP calls. (**B**,**F**) Agreement on Illumina InDel calls. (**C**,**G**) Agreement on Oxford Nanopore (ONT) SNP calls. (**D**,**H**) Agreement on ONT InDel Calls. For each figure, the bars indicate the number of variants called by the groups, indicated by filled circles below, across the whole dataset.

To determine the consistency in variant calling across workflows before and after ALTDP-, DP-, and AF-based filtering, the workflow calls were compared and plotted as upset plots (Figure 4). (Figure 4A–D) demonstrate the level of agreement before any filtering was applied to the seven workflows, with only variants with an AF of at least 15% considered, and with SPDI and Parsimony normalizations applied. Across the seven workflows, Illumina data showed strong agreement, even prior to filtering for both SNPs and indels (Figure 4A and Figure 4B, respectively). However, for the five workflows that analyzed the ONT data, the SNP and indel (Figure 4C and Figure 4D, respectively) results were dominated by single workflow’s unique calls. Across the Illumina and ONT data, (Figure 4E and Figure 4G, respectively) filtering increased agreement in the SNP calls across the workflows, with the fraction of calls found by all but one workflow increased from ~25% to over 86%, and the fraction found only by a single workflow decreased from over 60% to just over 2%. Thus, the majority of workflow discrepancies can be attributed to calls with AF < 50%, calls at locations with DP < 100 or an ALTDP < 50, calls from samples with poor reference genome coverage (<50%), calls in homopolymer regions, and/or from single-end data. While the approach did improve the agreement in indel calls for both Illumina and ONT (Figure 4F and Figure 4H, respectively), with the fraction of calls found by all but one workflow increasing from ~2% to over 64% and the fraction found only by a single workflow decreasing from over 91% to over 22%, the extent of agreement was not as extensive as that seen for SNPs. Notably, the total number of indel calls was greatly reduced to ~1.5% of the unfiltered count, suggesting that the majority of indel calls may be artifactual or otherwise poorly supported. In support of this, the large number of workflow unique calls for EDGE-COVID19 and BEI in Figure 4C and Figure 4D, respectively, are attributable to calls with low read support (DP < 100 or ALTDP < 50), of low frequency (<50%), as indicated by the reduction after filtering, Figure 4G,H, respectively. The initial test set also showed agreement in variant calls post-filtering, with the fraction of SNP calls found by all but one workflow increasing from ~86% to over 92% and the fraction found only by a single workflow decreasing from over 5% to ~1.5% (Supplementary Figure S2). Finally, the agreement in both SNPs and indels calls across the length of the genome was examined (Supplementary Figure S3), and no significant association between workflow disagreement and any portion of the genome was observed as evidenced by the relatively even distribution of deep red and deep blue color along the y axis (genomic position). Thus, consistent results across divergent deep sequencing workflows can be achieved with a combination of input data cleaning and algorithm parameter-based filtering.

**Figure 5:**
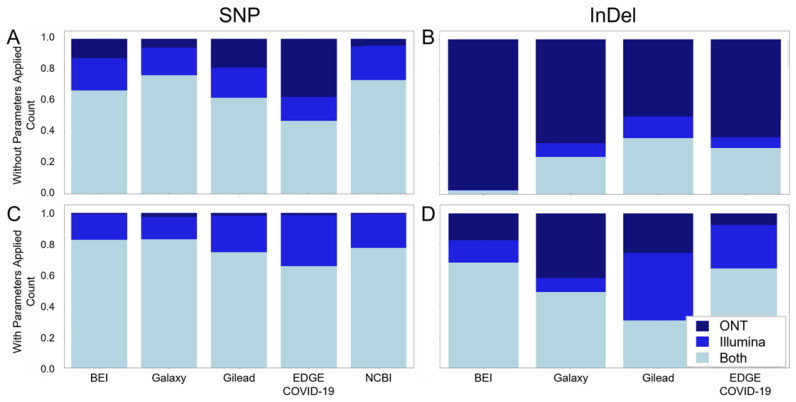
Application of recommended parameters results in increased agreement across platforms. Graphical representation of the agreement between platforms without the application of recommended parameters of SNP (**A**) and InDel (**B**) calls. (**C**) (SNP) and (**D**) (InDel) represent the agreement between platforms after the application of the recommended parameters. For each figure, only those samples for which both Illumina and ONT platform data had at least one variant call that passed all of the filters were considered. The total height is normalized to the total number of calls made by each workflow, with light blue portion indicating calls made on both platforms for a given sample, medium blue indicating calls made only for Illumina data, and dark blue indicating calls made only for ONT data.

Similarly, to assess the extent of agreement and disagreement across sequencing platforms, stacked bar plots were generated (Figure 5), with samples being considered only if both Illumina and ONT data passed the filtering criteria described above. Similar to what was seen with the cross-workflow comparison, the results for both shared SNPs and indels without filtering (Figure 5A,B, respectively) were markedly improved for both SNPs and indels with the application of our filters (Figure 5C,D, respectively), with nearly no ONT-unique SNP calls and an increase in the number of SNP calls common between platforms of 10–20%, depending on the workflow, after filtering. Indel agreement was similarly improved, with an increase in the percent of common calls of up to 60%, depending on the workflow. Again, similar to what was seen with the cross-workflow comparison, the initial dataset showed similar agreement for SNPs and more modest agreement for indel calls post-filtering (Supplementary Figure S4), suggesting factors others than those considered in the filtering account for discrepancies in indel calls earlier in the pandemic. 

**Figure 6:**
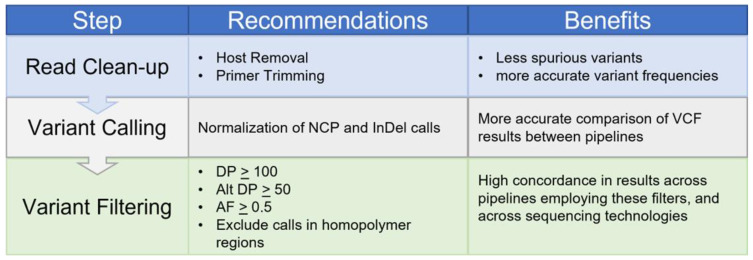
Variant calling workflow recommendations. Outline of the recommendations for each step in a variant calling workflow, from read cleanup to variant filtering, are illustrated. Additionally, the benefit of implementing the recommendations at each step are noted.

In summary, these findings underscore the fact that variant calling data provided by diverse workflows are not consistent, unless the processes by which the sequences were analyzed reflect best practices in variant calling for SARS-CoV-2. To that end, the filtering we identified and summarized in Figure 6 provides a robust playbook for diverse variant calling workflows for both Illumina and ONT platform data, and supports robust agreement between these platforms. To briefly summarize, host sequences need to be removed and primers trimmed; a minimum depth of 100, a minimal alternate allele support of 50, and a minimal allele frequency of 50% should be used when generating consensus sequences to maximize interoperability of the resulting sequences across both workflows and sequencing technologies. Additionally, variants identified through low-quality sequence data with poor reference sequence coverage or calls in homopolymer regions should be carefully scrutinized, as workflows can vary significantly in their results under these conditions. These recommendations, if adopted, should support consistent reporting of consensus sequences for downstream analyses, and the fair comparison of results generated by many different sequencing groups.

## 3. Discussion

Over the course of the pandemic, genetic surveillance focus shifted from the analysis of consensus genomes alone to include more frequent use of the underlying sequence data. While consensus genomes reduce the complexity and size of the data, recent cases of recombinants [26,27], co-infection [28,29], and monitoring for drug resistance has made clear the importance of working beyond the consensus genome. If only the consensus sequence is provided, contamination that may have occurred during sample collection, laboratory preparation, sequencing, or bioinformatics processing may be interpreted as a coinfection. In absence of corresponding raw data, these potentially confounding factors cannot be easily tracked and corrected. However, as consensus genomes are usually the starting material for many common analyses, we aimed here to identify factors which influence the accuracy and consistency of variant calling results.

The importance of primer trimming, shown in Figure 1, highlights the need for comprehensive metadata when sharing data publicly, most notably information on which primer scheme was used. While consensus between platforms is possible, as illustrated by Figure 5, the potential challenges highlight the need for transparency regarding how the original sequencing was performed, even if the submitted sequence is a consensus sequence; specifically, information on what instrument was used for sequencing, along with the method and parameters used for consensus construction. Additionally, the difficulty in finding consensus with low-quality data suggests a need for quality standards for SARS-CoV-2 NGS data. One such approach would be to identify a minimum reference coverage and a minimum average depth of coverage, such that data below the minimum threshold cannot yield consistent-enough results to be used for common downstream analysis, such as lineage assignment or phylogenetic reconstruction.

Given the number of discrepancies found even when only considering a minimum within-sample allele frequency of 15% and the parameter setting found necessary to ensure inter-workflow compatibility, the use of minor alleles should be considered carefully. In support of this suggestion, prior work on respiratory syncytial virus sequencing found that, below allele frequencies of 25%, variant calling across a range of coverages, variant callers, and a range of simulated error profiles resulted in a precision of less than 50%, approaching 0% at allele frequencies below 5% [30]. Additionally, sequencing analysis on influenza samples has found that minor alleles cannot be confidently called at frequencies below 5% [31], and that without correction to the methods, minor variants were overestimated by as much as 10-fold [32]. These studies also identified several methods to improve confidence in variant calls, including generating technical replicates for each sample and adjusting allele-frequency thresholds based on Polymerase Chain Reaction (PCR) cycle threshold values, both of which underscore the need for rich metadata to support thorough and accurate use of public sequence data and the need to consider consensus sequences carefully in the absence of such metadata. Furthermore, these suggestions highlight a topic not examined in this work, which is the need to carefully consider wet-lab controls to ensure quality data are generated prior to bioinformatic analyses. Together with the results presented here, this suggests that more research is needed into the conditions under which minor variants (<15% allele frequency) can be determined most reliably.

Pharmaceutical and biotechnology companies are required to comply with Good Practice (GxP) guidelines and obtain system validation when developing computer software to ensure that medical devices, drugs, and other life science products are safe and effective [33]. At present, it is unclear what level of computer system validation is required by regulatory agencies, including the FDA for NGS analysis workflows, and regulatory agencies typically perform their own independent analyses and compare them to the submitted analysis from sponsors. Given the numerous sample preparation methods and sequencing platforms, as well as the various analysis workflows (many of which are proprietary), this presents challenges for regulatory review of deep sequencing data [34]. In the future, the FDA anticipates the development of standardized analysis workflows that will provide reproducible data analysis sufficient for generating consistent and robust results from NGS data [34]. However, we have shown that standardized deep sequencing analysis workflows are currently not used across academia, government, and industry. Further collaborative efforts, like the work from ACTIV TRACE shown here, are needed to achieve the standardization of workflows and reproducibility of results that are needed in the face of pandemic threats. This collaboration representing industry, government, and academic partners has moved us closer to the standardized analysis that will be required for deep sequencing, suggesting that regulatory agencies should implement these robust recommendations in their current guidelines.

## 4. Methods

### 4.1. Dataset

RefSeq [35] accession sequence NC_045512.2 was used as reference genome for alignment for all but one workflow, Lilly‘s, the sequence for which is 99% identical, with zero gaps, by Needleman–Wunsch alignment. A description of the data analyzed in the original and recent datasets can be found in Supplemental Tables S2 and S3, respectively. Briefly, Dataset 1 consists of 413 sequence records, representing 155 samples, while the recent set, Dataset 2, consisted of 419 sequence records, representing 210 samples. Dataset 1 generally represents the pre-Alpha lineages with some Alpha, while Dataset 2 has a mixture of Alpha, Delta, and Omicron lineages. Both sets are constituted by paired Illumina and ONT samples. The records were processed by each of seven workflows described below. The results were then combined, and SNPs were normalized using the SPDI algorithm [24]. Subsequently, the InDel results are normalized using the Parsimony script, described below, and the additional analyses and figures were generated via python scripts, all available here (https://github.com/ncbi/ACTIVTRACEvariants, accessed on 4 December 2023).

### 4.2. BEI Resources

SARS-CoV-2 reads were retrieved from NCBI’s [36] Sequence Read Archive (SRA) directly using the fastq-dump v2.11.1 utility (https://github.com/ncbi/sra-tools, accessed on 4 December 2023). These reads were then trimmed and filtered to remove adapter sequences and low-quality reads, using *fastp* [37] v0.23.2 for Illumina reads and *NanoFilt* [38] v2.8.0 for Oxford Nanopore reads. Settings for *fastp* were left at default, while *NanoFilt* was set for a minimum average quality of 10 and minimum length of 150, while trimming the first 30 bases of each read. Following this, both sets of reads were taxonomically binned using ATCC’s *bin_reads* function, which relies on *kraken2* [39] v 2.1.2 to identify the nearest taxonomy of any given read. Kraken2 classification was run with default settings and its *bacterial_viral_db* database. Kraken2 also contains an extract reads function that was employed using NCBI: taxID694009, which corresponds to SARS-Coronavirus and all sub-taxa.

All Illumina reads that have been processed as outlined above were entered into an identical workflow for variant calling analysis. This workflow was comprised of four steps: read mapping to NC_045512.2 (Wuhan-Hu-1), local realignment, variant calling, and normalization. Prior to alignment, paired-Illumina fastqs needed another step of processing. Because kraken2’s taxonomic binning does not guarantee that both forward and reverse fastqs will have the same resulting reads, *seqkit common* [40] v 2.1.0 was used to ensure that only paired reads are further analyzed. Alignment was performed with *bwa* mem [41] v 0.7.17-r1188 using default parameters. Local realignment began with *bcftools mpileup* [42] v1.12 to produce a per-base pileup to stage the realignment. This used default settings, except for the max-depth, which was set to 8000 to reduce the chance of missing minority variants due to excessive depth at a position. Next, *bcftools call* and *bcftools filter* were used to capture multiallelic variants with a variant call quality above 30. This initial vcf was passed to GATK [43] v4.2.2.0 to apply base quality score recalibration within the context of all bases at each position. Finally, realignment was achieved using the *lofreq Viterbi* [44] tool v2.1.5, using default parameters. Variant calling was performed using *lofreq* v2.1.5 in four stages: *indelqual* with “--dindel” parameter and *alnqual* to capture indel variants, *call* with --call-indels and -C 50 to capture all variants above a depth of 50×, and *filter* with -v 50 and -a 0.15 to further filter for minimum depth of 50× and alternate allele frequency of 15%. 

Oxford Nanopore long reads have a few fundamental differences that affect what off-the-shelf tools can be used for their processing. Namely, underlying algorithms in *fastp*, *GATK*, and *bwa mem* are not designed for the increased length and error profile of these reads. Consequently, medaka_haploid_variant [45] v1.5.0 was used to perform the initial alignment, and its BAM file was passed to lofreq in the same manner as in the Illumina dataset. Minimum depth and alternate frequency filters were applied afterward, and the previously described step of taxonomic binning was identical to the Illumina dataset.

### 4.3. Galaxy Project 

Illumina short-read (paired-end subset of the data only) and Oxford Nanopore long-read data was downloaded in fastq.gz format from the FTP server of the European Bioinformatics Institute (EMBL-EBI) at ftp.sra.ebi.ac.uk (accessed on 22 May 2022). Variant calling was then performed with platform-specific Galaxy workflows [46], which have previously been described [47] and are publicly available from Dockstore [48] and the WorkflowHub [49], against NCBI Reference Sequence NC_045512.2. No attempt was made to analyze single-end Illumina data. A brief overview of the analysis of data from both platforms is provided below.

#### 4.3.1. Variant Calling from Paired-End Illumina Short-Read Data

We used fastp (version 0.23.2) for read trimming and quality control [38], aligned reads with bwa-mem (version 0.7.17) [41], filtered for reads with fully mapped read pairs (with samtools, version 1.9 [42]), re-aligned reads using the lofreq viterbi command from the lofreq package (version 2.1.5 used here and in all subsequent steps using lofreq) [44] and calculated indel quality scores with lofreq indelqual. We then attempted to trim amplification primers from the aligned reads using ivar trim (version 1.3.1) [50] assuming the ARTIC v3 primer scheme [51] had been used in amplification of all samples (which is true for the majority of the samples analyzed, but not all of them; we continued with untrimmed data for those other samples). The remaining, trimmed alignments served as the input for variant calling with lofreq, and variant calls down to an allele-frequency of 0.05 were reported if confirmed by at least 10 reads.

#### 4.3.2. Variant Calling from Oxford Nanopore Long-Read Data

We used fastp (version 0.23.2) for quality control [37] and read length filtering. For samples that we detected to be amplified with the ARTIC v3 primer scheme we filtered for read sizes between 300 and 650 bases, for other samples we allowed read sizes between 300 and 3000 bases. Retained reads were aligned with minimap2 (version 2.17) [52], and successfully mapped reads left-aligned with BamLeftAlign from the freebayes package (version 1.3.1) [53], after which we attempted to trim primers from the reads with ivar trim (version 1.3.1), again assuming the ARTIC v3 primer scheme and using untrimmed data where that assumption failed. The data was then analyzed with the medaka consensus tool (https://github.com/nanoporetech/medaka, accessed on 4 December 2023, version 1.0.3) [45] and variants extracted with medaka variant tool (version 1.3.2) and postprocessed with medaka tools annotate (integrated into the medaka variant tool’s Galaxy wrapper; https://toolshed.g2.bx.psu.edu/repository?repository_id=a25f9bf8a7d98ae4&changeset_revision=0f5f4a208660, accessed on 4 December 2023, 09/20/21 installation). Variant calls down to an allele-frequency of 0.05 were reported if confirmed by at least 10 reads and called, in the case of SNVs only, with a QUAL score of at least 10. 

### 4.4. Gilead Sciences

Illumina short-reads and Oxford Nanopore long-read data were downloaded from Sequence Read Archive (SRA) using sratoolkit (https://github.com/ncbi/sra-tools#the-sra-toolkit, accessed on 4 December 2023, v 2.8.1).

#### 4.4.1. Illumina

Fastq files were aligned to hg38 reference using BWA v0.7.15 [41] to exclude human RNA transcripts and to isolate viral reads for further processing. Next, reads were trimmed using Trimmomatic v0.36 [54] for low quality (sliding window 4 bp, avg phred 15) and short reads (<50 base pairs) were filtered out. Paired end reads that overlap were merged using NGmerge v0.3 [55] software, creating a single-end fastq file containing merged reads and any single end reads that do not overlap. Reads were then aligned to the Wuhan-Hu-1 reference (NC_045512) using SMALT v0.7.6 aligner (https://www.sanger.ac.uk/tool/smalt-0/, accessed on 4 December 2023). If amplification primer information was available, trimmed the base pairs from reads that overlap with primers. Tabulate nucleotide variants and indels per genome position (NC_045512), excluding any variants with average phred score less than 20 and read depth less than 50, as well as any frameshift indels.

#### 4.4.2. ONT

Fastq files were aligned to hg38 reference using minimap2 v2.17 [56] to exclude human RNA transcripts and to isolate viral reads for further processing. Reads were then aligned to the Wuhan-Hu-1 reference (NC_045512) using minimap2 v2.17 [56]. If amplification primer information was available, trimmed the base pairs from reads that overlap with primers. Tabulate nucleotide variants and indels per genome position (NC_045512), excluding any variants with average phred score less than 10, forward strand ratio < 0.1 or > 0.9, and read depth less than 50, as well as any frameshift indels. 

### 4.5. Los Alamos National Laboratory (EDGE-COVID19)

Illumina short-read and Oxford Nanopore long-read data were downloaded from Sequence Read Archive (SRA) using sratoolkit (https://github.com/ncbi/sra-tools#the-sra-toolkit, accessed on 4 December 2023, v 2.9.2). Quality control, read mapping, variant calling, and consensus genome generation were then performed with EDGE-COVID19 (EC-19) workflows (http://edge-COVID19.edgebioinformatics.org/, accessed on 4 December 2023, v20220427). Detailed description of the EC-19 workflows has also been previously described [19] (https://edge-COVID19.edgebioinformatics.org/docs/EDGE_COVID-19_guide.pdf, accessed on 4 December 2023). Briefly, the EC-19 workflow employs many commonly used tools such as FaQCs (v2.09) for quality control, Minimap2 (v2.17, default for ONT) or BWA mem (v0.7.12, default for Illumina) for mapping reads to a SARS-CoV-2 reference genome (NC_045512.2 without the 33nt poly-A tail in the 3′ is used as default), an algorithm based on ARTIC workflow (https://github.com/artic-network/fieldbioinformatics, accessed on 4 December 2023) for trimming primers if an amplicon-based method (e.g., ARTIC, SWIFT) was used for sequencing, generating consensus genomes, and variant calling based on samtools mpileup (v1.9) wrapped into a custom script (https://gitlab.com/chienchi/reference-based_assembly, accessed on 4 December 2023). EC-19 then accounts for strand biasness using both fisher score and Strand Odds ratio (https://gatk.broadinstitute.org/hc/en-us/articles/360036464972-AS-StrandOddsRatio, accessed on 4 December 2023) and reports SNVs if the Allele Frequency (AF) > 0.2 and has a minimum Depth of Coverage (DC) of 5. Likewise, InDels are reported if DC > 5, and then platform-specific thresholds are implemented for AF, as AF > 0.5 is required for Illumina and AF > 0.6 for ONT data in order to account for the higher error rates with this platform, and >0.8 within homopolymer sequences [57,58].

### 4.6. Lilly

#### Illumina

The paired end raw sequencing data were trimmed in two successive rounds using cutadapt [59] version 2.5 with the following parameters: pe1_parms: --quality-base = 33 -a ‘G{150}’ -A ‘G{150}’: pe2_parms: -a AGATCGGAAGAGCACACGTCTGAACTCCAGTCAC -A AGATCGGAAGAGCGTCGTGTAGGGAAAGAGTGTAGATCTCGGTGGTCGCCGTATCATT: --quality-base = 33 -q 20 -n 2 --trim-n -m 20 --max-n = .2: -a ‘A{150}’ -A ‘A{150}’ -a ‘T{150}’ -A ‘T{150}’: -g ‘A{150}’ -G ‘A{150}’ -g ‘T{150}’ -G ‘T{150}’: -g AAGCAGTGGTATCAACGCAGAG -G AAGCAGTGGTATCAACGCAGAG: -g AAGCAGTGGTATCAACGCAGAGTAC -G AAGCAGTGGTATCAACGCAGAGTAC. Reads were aligned to the hg19_human_trxome_and_coronavirus.fa reference genome (Thermo Fisher, Waltham, MA, USA, which included MT019532.1 BetaCoV/Wuhan/IPBCAMS-WH-04/2019) using bwa-mem [41] version 0.7.12 with default parameters. Variants were called using FreeBayes [53] version 1.3.1 with the following parameters: -F 0 -p 1 -K -C 0 -n 5 -w --min-alternate-count 0 --min-alternate-fraction 0. Variants were reported if there were ≥5 reads supporting the variant; if ≥10% of reads supporting the variant were derived from the minor strand; and if the allele frequency was ≥15%. 

### 4.7. NCBI 

#### 4.7.1. Illumina

Illumina short reads were downloaded from SRA database using fastq-dump of sratoolkit (https://github.com/ncbi/sra-tools#the-sra-toolkit, accessed on 4 December 2023, version 2.11.0), then trimmed using trimmomatic (version 0.39) [54]. The reads were aligned using Hisat2 (version 2.2.1) [60], then left-aligned using GATK LeftAlignIndels (version 4.2.4.1) [61]. GATK HaplotypeCaller (version 4.2.4.1) with the options “minimum-mapping-quality 10” [62] was used for generating variant VCFs with NCBI Reference Sequence NC_045512.2 as the reference. Calls with QUAL value smaller than 100, alternate allele counts lower than 10, FS value smaller than 60, SOR value smaller than 4, QD value equal to or greater than 2, ReadPosRankSum value equal to or greater than −4, allele frequency lower than 0.15, and reference genome positions beyond 29,850 were excluded. 

#### 4.7.2. ONT 

Nanopore reads were downloaded from the SRA database using fastq-dump of sratoolkit (https://github.com/ncbi/sra-tools#the-sra-toolkit, accessed on 4 December 2023, version 2.11.0), then trimmed using NanoFilt [38] (version 2.8.0) with the options “q 10” and “headcrop 40” [38]. Two rounds of medaka (version 1.3.2, https://github.com/nanoporetech/medaka, accessed on 4 December 2023) were applied to generate consensus assembly. Consensus to reference (NC_045512.2) alignment and initial variant calls were generated with MUMmer (version 4.0.0rc1, https://github.com/mummer4/mummer, accessed on 4 December 2023). InDels and SNPs within 10bps of an InDel were excluded using bcftools (version 1.11) [43], and snp clusters (2 or more SNPs within 10 bps) were filtered using vcftools (version 0.1.12b) [62].

### 4.8. VIR

#### 4.8.1. Illumina

The workflow was written and optimized for another library preparation, and the primer removal step and workflow parameters were not optimized for the samples analyzed in this study. Illumina short reads were downloaded from the SRA database using fastq-dump of sratoolkit (https://github.com/ncbi/sra-tools#the-sra-toolkit, accessed on 4 December 2023, version 2.9.1) [parameters: *–split-files* for paired end reads]. The library preparation consisted of a mix of SE reads and PE reads (with random fragmentation or amplicons fragment). The read length ranged from 300 to 500bp for SE, and 50 to 300bp for PE reads. As the samples were prepared with different library preparations, not always retrievable from the metadata, we used a conservative approach to trim the 31bp at the beginning of all reads that were 150bp or longer; 31bp corresponds to the maximum primer length in the ARTICV3 kit. Reads were trimmed with trimmomatic (version 0.39) [parameters: HEADCROP:31 MINLEN:35; PE -validatePairs for paired end; SE for single end] [54]. The alignment was performed with bwa-mem (quay.io/biocontainers/bwa: 0.7.17--hed695b0_7) [parameters: -M] [63]. The variant calling was performed with lofreq (quay.io/biocontainers/lofreq:2.1.5--py36ha518a1e_1) [44] in multiple consecutive steps: lofreq viterbi; lofreq indelqual [parameters: --dindel]; lofreq call-parallel [parameters: --no-default-filter --call-indels –min-bq 6 –min-alt-bq 6 –min-mq 1 –sig 1]; lofreq filter [parameters: --no-defaults –af min 0.01 –cov min 15 –sb-mtc fdr –sb-alpha 0.05 –sb-incl-indels; note: --sb-alpha parameter was set to 0 for samples prepared with amplicon libraries to prevent filtering variant due to strand bias, which can occur with amplicon library preparation]. For each sample, SNVs present at AF > 0.5 were substituted in the reference genome with bcftools consensus (quay.io/biocontainers/bcftools:1.10.2--hd2cd319_0) [42], and the alignment and variant calling steps were run a second time on the “new” reference genome (in order to rescue reads that were potentially mis-aligned/un-aligned due to too many mutations located in the near vicinity of each other). Of note, the final variant calling was done with regard to the initial NC_045512.2 reference genome nomenclature. SNVs and indels were reported if they were present with AF > 0.15. Minimum read depth was set at a low threshold (15 reads) for the purpose of this analysis. Variants flagged as potentially spurious due to their location on the reads (i.e., consistently located at the same position in the read for the alternative allele, but not for the reference allele) were further filtered if they were present in more than 2 samples and in all samples at low AF (<0.5). 

#### 4.8.2. ONT

At the time of analysis, VIR did not have an existing workflow to assess ONT data. 

## 5. Parsimony Script

Following SPDI-normalization, the parsimony script was applied. After pulling the SPDI-processed data into a data table, the first step was to sort data according to analytical group, sample accession, and position. Then, it must resolve adjacent indels into singular records. Where InDels used a hyphen (-) to represent the reference or alternate allele, we searched for any adjacent rows with identical group and accession values, and the positions were consecutive. For each record that satisfied these requirements for either the next or previous record, they were grouped together and concatenated according to the Group and Acc values, with any number of repeated hyphens being replaced with a singular hyphen to match SPDI formatting. This only saved the depth and alternate allele values for the first record in the set of each consecutive InDel. In practice, we have not found this disruptive to our analysis, but this may present an issue when this occurs closer to the limit of detection or when precise depth is significant. 

Next, we must address the nucleotide context issue. This combined data table was looped over, row by row, capturing the assorted metadata in each row. These fields will not be altered. We are only concerned with InDels that do not conform to our simple formatting. First, we checked for the simple cases that either the reference or alternate allele completely contains the other, such as AAA4313AAAA. In this case, we removed the shorter of ref or alt from the other, and replaced the shorter field with a hyphen (-) and write to file, transforming AAA4313AAAA to A4313-. 

This left the more complex case of a single record containing both an InDel and an SNP. These records have neither a reference nor alternate allele that completely contains the counterpart, so we could not simply remove the identity of one from the other, as we had before. Instead, we aligned the reference allele and the alternate allele for each qualifying row using biopython’s [64] pairwise2 module as align.globalms with settings match = 2, mismatch = −0.5, gapopen = −1, gap extension = 0.1, and allowed only the top alignment. These settings were determined to be sufficient for the data presented in this study, but have not been optimized to other datasets. For each alignment, we determined where a deletion may be found and saved InDel in the ‘type’ field of the metadata variable. For all other nonmatching bases in the alignment, we saved SNP to the ‘type’ field of the metadata. In both cases, the position of the variant was incremented by the number of preceding matching bases to correct for where the variant occurs. These explicit and separated InDel and SNPs were written as discrete records to file. In all remaining cases, both the reference and alternate allele values match with at least the starting nucleotide of the record. We removed all matching alternate alleles from the reference alleles that had not been otherwise addressed from the beginning of the reference, and incremented the position by the length of the removed bases. This left only a deletion in all observed cases. These were written to file with a hyphen in place of the alternate allele. 

## 6. Studying the Effects of Primer Trimming on Variant Calls and Apparent Allele Frequencies of Called Variants

In total, 83 high-quality ARTICv3-amplified samples were selected from the ACTIV TRACE Illumina paired-end sample collection. These samples were analyzed twice with the Galaxy workflow, once with and once without primer trimming. The position of each resulting variant call (2696 calls total with, 2637 without trimming) was compared to the known primer binding sites of the ARTICv3 primer scheme to classify calls as inside (421 calls with, 360 calls without primer trimming) and outside of primer binding sites. For variants called with and without primer trimming, the observed variant allele-frequency with primer trimming was plotted against the same metric without primer trimming. For variants called only with primer trimming, a value of zero was used as a substitute for the unobserved variant allele-frequency without primer trimming.

## 7. Calculation of Receiver Operating Characteristic (ROC) Plots Based on Concordance across Workflows

For each participating group, all variant calls made for all samples analyzed with the group’s workflow were classified as either concordant or discordant based on whether that same variant had or had not been called for the same sample by the majority of groups (≥6 workflows) that had analyzed that sample. For the purpose of generating ROC-like plots, concordant and discordant calls were treated as true-positive and false-positive calls, respectively. The true- and false-positive lists of each group were then filtered independently, with increasing thresholds on two key variant call metrics: the number of sequencing reads supporting the variant allele (alternate allele read depth, AltDP) and the fraction of all sequencing reads at the variant site that support the alternate allele (alternate allele-frequency, AF). Increasing thresholds of each of the two metrics lower the number of true- and false-positive calls, but to different extents. For plotting, the numbers of retained true- and false-positive calls at each threshold were normalized to the numbers of unfiltered true- and false-positive calls of the respective group, thus the ROC AUCs cannot be directly compared between groups.

## 8. Calculation of Receiver Operating Characteristic (ROC) Plots Based on Cross-Platform Agreement

For samples for which both Illumina and ONT sequencing data were available, alternative plots could be generated as follows: for each participating group, all variant calls made for all samples analyzed with the group’s workflow based on the data for one of the two sequencing platforms were classified as concordant or discordant based on whether that same variant had or had not been called for the same sample on the other platform either by any participating group or not. For plotting, the true- and false-positive lists of each group resulting from this alternate classification were used to create a threshold, and were subsequently normalized as described above.

## Data Availability

Code and data are available from https://github.com/ncbi/ACTIVTRACEvariants/ (accessed on 4 December 2023).

## Supplementary Materials

The following supporting information can be downloaded at: https://www.mdpi.com/article/10.3390/v16030430/s1, Figure S1: Indel Calls across the length of the SARS-CoV-2 Genome.; Figure S2: Agreement across workflows with and without recommended parameters, recent dataset; Figure S3: Difference in variant call frequencies across the length of the reference genome for each workflow; Figure S4: Agreement across platforms with and without recommended parameters, recent dataset. Table S1: Workflow Details; Table S2: Dataset 1 Metadata; Table S3: Dataset 2 Metadata.
