# Supplementary figures and images for: Recommendations for Uniform Variant Calling of SARS-CoV-2 Genome Sequence across Bioinformatic Workflows
Source: Viruses. 2024 Mar 11;16(3):430. doi: 10.3390/v16030430 (PMC10975397; doi:10.3390/v16030430)

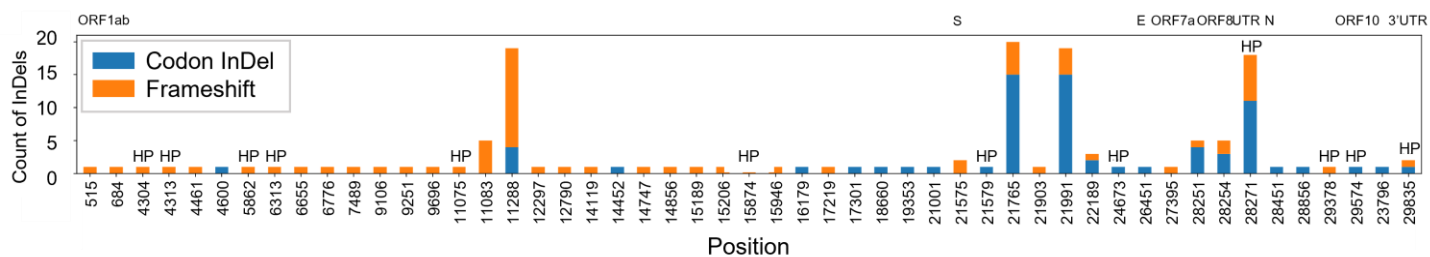

Supplement: Supplementary file 1 [file viruses-16-00430-s001.zip › DSAS SFigure 1.pdf]

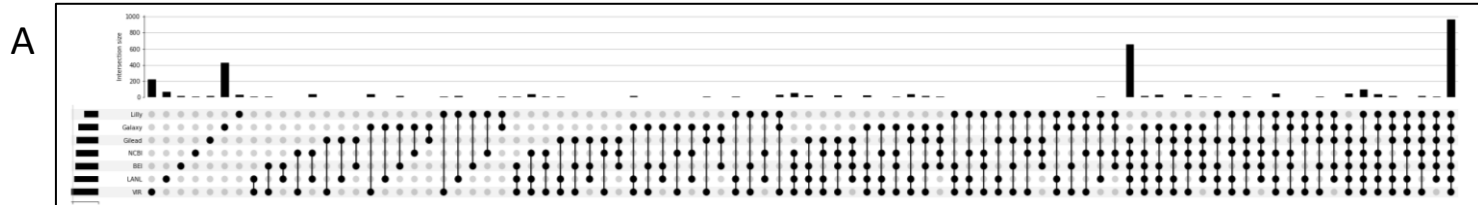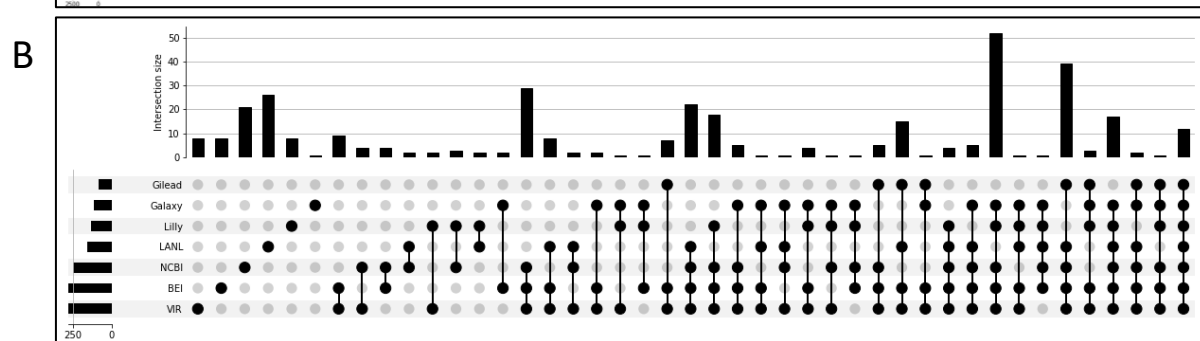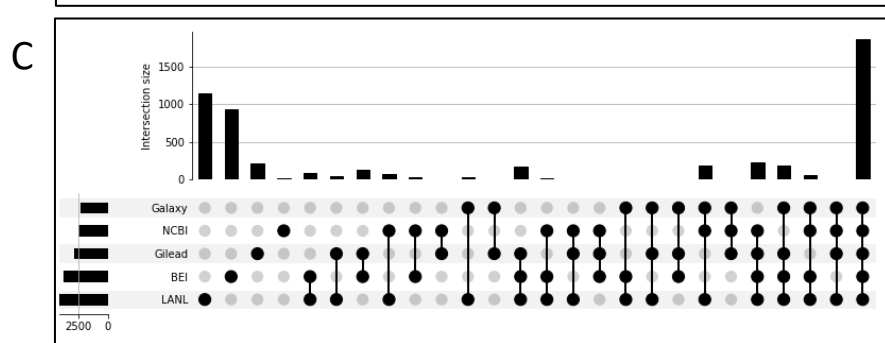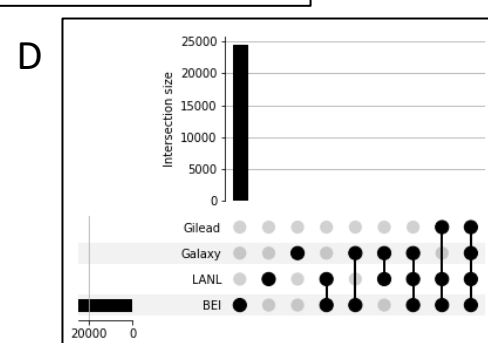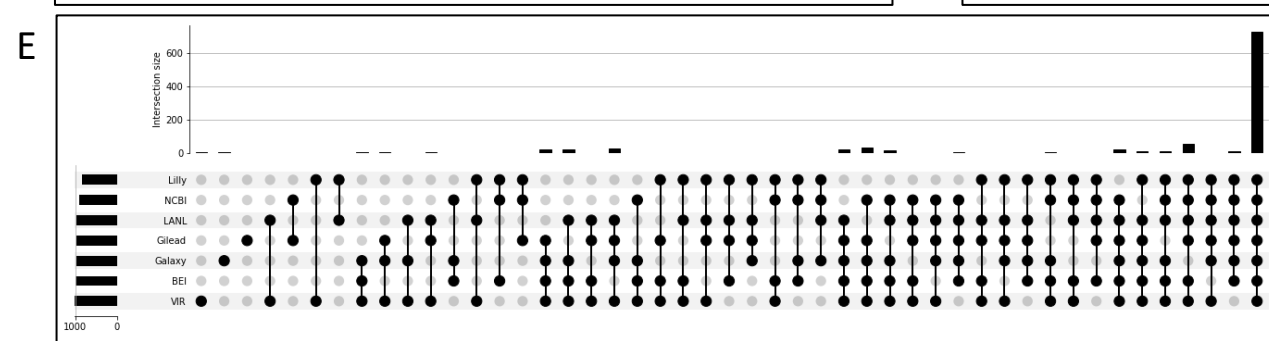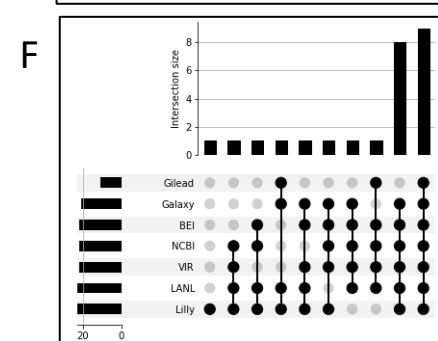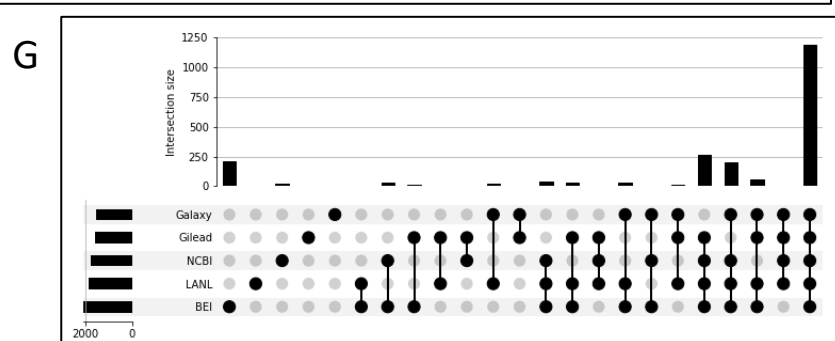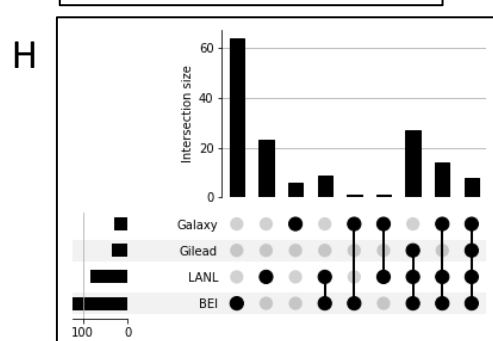

Supplement: Supplementary file 1 [file viruses-16-00430-s001.zip › DSAS SFigure 2.pdf]

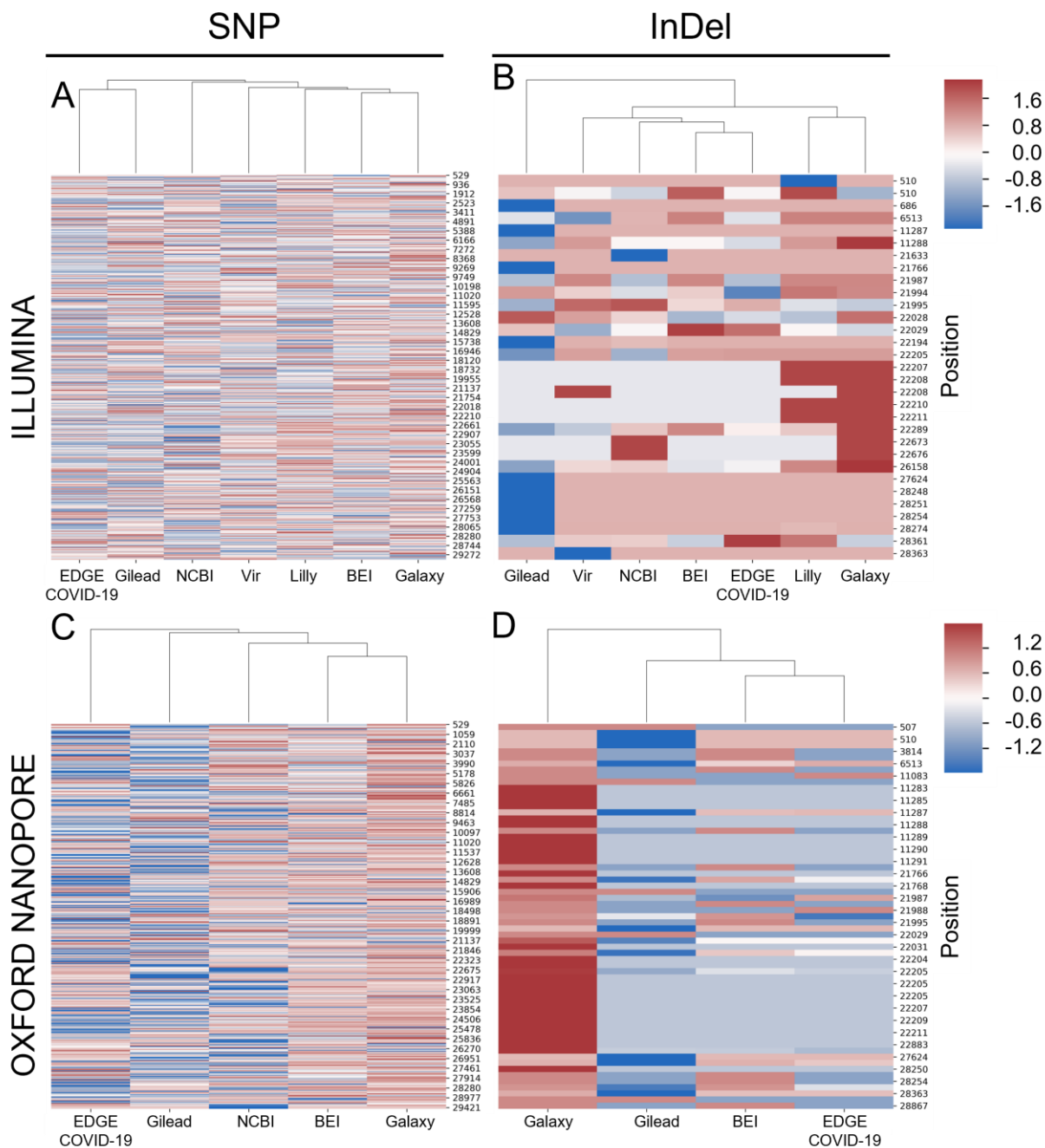

Supplement: Supplementary file 1 [file viruses-16-00430-s001.zip › DSAS SFigure 3.pdf]

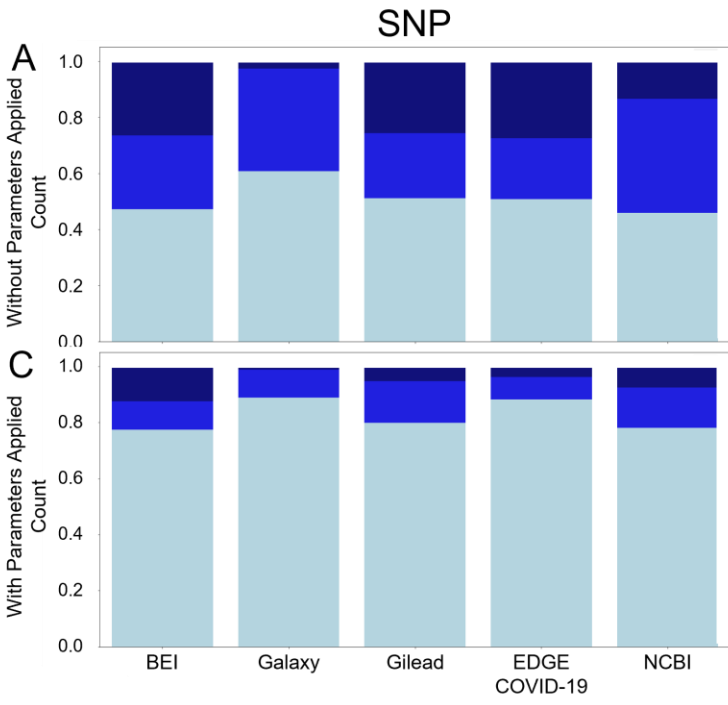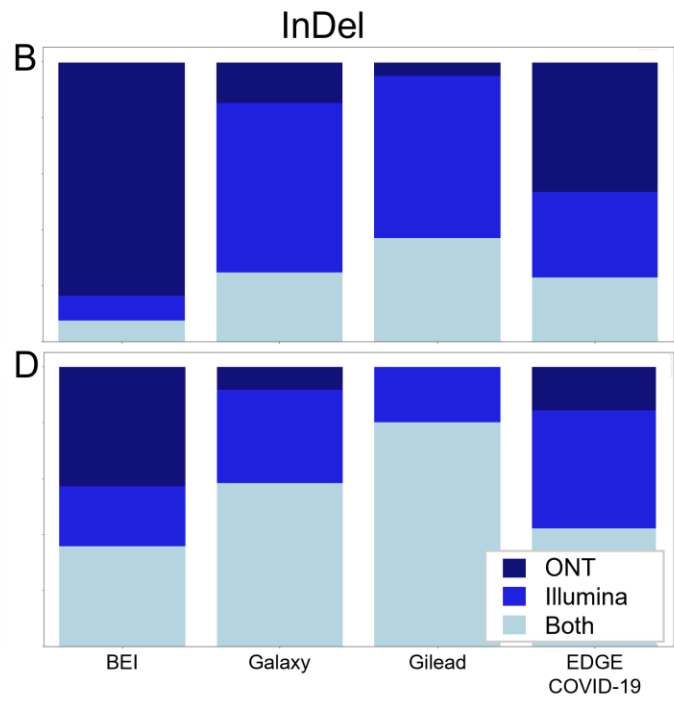

Supplement: Supplementary file 1 [file viruses-16-00430-s001.zip › DSAS SFigure 4.pdf]
